# 

**Published:** 1917-10-20

**Authors:** 


### CONTENTS.

| | PAGE |
|---|---|
| **National Service Under New Management** ... | 41 |
| **Why British Public Sanatoria Have Failed** ... | 42 |
| **Hospital and Institutional News** ... ... ... | 43 |
| Sir Alfred Keogh, G.C.B., M.D., F.R.C.S.—Orthopædic Hospitals for Soldiers—The American Ambassador at Leeds—The Cardiff Hospital Deficit—The Power of the Penny—A Medical Board's Action—The Birmingham Saturday Institutions—Bumble in Uniform—Orthopædics Sixty Years Ago—The Absence of Standard in Small War Hospitals—Preparing for Air-raids in Hospitals—Sunderland Schools Medical Officer—Have General Practitioners' Fees Risen?—Meat Inspection—The Value of Tuberculin—Ophthalmia Neonatorum—Dependants of Soldiers in Rate-aided Institutions—Sailors' and Soldiers' Home—More Beds for Salford?—An Offer which has made History—*The National Food Journal*—The Sanatorium Treatment Collapse—Consumption at Gainsborough—Curious Patriotism—War Bonuses for Asylum Workers—Mrs. Atkins Writes Again—The First Annual Subscriber—Results of Baby Week—Lincoln Mothercraft School—This Week's Drug Market. | |
| **The Military Position of the Medical Student in Great Britain and in America** ... ... | 49 |
| A Contrast in Policy and Methods. | |
| **The Report of the Cinema Commission** ... | 51 |
| A State Censorship Favoured. | |
| **The Editor's Letter-Box** ... ... ... ... | 53 |
| "Raising a Ghost it were Better to Lay"—North Staffordshire Infirmary: The Way to get Money—"He was Bought a Bun"—The Royal British Nurses' Association. | |
| **The Matrons' and Sisters' Department** ... | 55 |
| The Suffrage and Florence Nightingale. | |
| Round the Hospitals. | |
| Miss L. V. Haughton's Striking Personality—Miss L. Clark Joins College Council—A New Theory of Woman's Fitness—The Splendid Service of Non-combatants—A Wondrous War Project—Importance of Home-made Jam. | |
| **Nursing Affairs in Ireland** ... ... ... ... ... | 59 |
| **Matrons' and Sisters' Appointments** ... ... | 60 |
| **The Institutional Worker** ... ... (Suppl.) | 1 |
| Starting a Venereal Block. | |

